# Osteoporosis resulting from acute lymphoblastic leukemia in a 7-year-old boy: a case report

**DOI:** 10.1186/1752-1947-8-168

**Published:** 2014-05-28

**Authors:** Hendra Salim, Ketut Ariawati, Wayan Bikin Suryawan, Made Arimbawa

**Affiliations:** 1 Division of Hematology and Oncology, Department of Child Health, Medical Faculty of Udayana University, Sanglah Hospital, Kesehatan Street No. 1, Denpasar, Bali, Indonesia; 2 Division of Endocrinology, Department of Child Health, Medical Faculty of Udayana University, Sanglah Hospital, Kesehatan Street No. 1, Denpasar, Bali, Indonesia

**Keywords:** Acute lymphoblastic leukemia, Children, Osteoporosis

## Abstract

**Introduction:**

Osteoporosis in children is rare and usually secondary to an underlying disease process whose diagnosis may be difficult to detect. Etiological factors responsible for osteoporosis secondary to chronic illness include immobility, pubertal delay and other hormonal disturbances. Rarely, it can be a manifestation of acute lymphoblastic leukemia. Most of the reported bone fracture incidences associated with acute lymphoblastic leukemia occur during the course of the chemotherapy, not at the point of the first symptoms of leukemic disease, as happened with the case presented here.

**Case presentation:**

A 7-year-old Asian Balinese boy presented with back pain. His anteroposterior pelvic radiograph showed osteoporotic bone. A bone age study revealed growth failure of his metacarpals, phalanges and sesamoid. His total bone mass density was 97% age-match. However, a peripheral blood smear showed normochromic anemia with thrombocytopenia. Immunophenotyping of his peripheral blood revealed no dominant markers, but a bone marrow aspiration confirmed a diagnosis of acute lymphoblastic leukemia.

**Conclusions:**

Osteoporosis was the only manifestation of the child’s underlying acute lymphoblastic leukemia. Leukemia was diagnosed when his bone marrow was found to contain more than 25% blasts. Because of leucopenia, the immunophenotype failed to reveal a dominant marker in this case, thus we were unable to classify the acute lymphoblastic leukemia.

## Introduction

Normal bone mass is defined by the World Health Organization as a bone mass density (BMD) within one standard deviation (SD) of the mean for young adults, osteopenia as increased bone loss with a bone mass between 1 and 2.5 SDs below normal, and osteoporosis as a bone mass ≥2.5 SDs below normal. There is no consensus about the definition of osteoporosis in pediatrics. Most bone specialists make a diagnosis of osteoporosis in children and adolescents only when BMD is low and there is at least one fracture [1,2].

Childhood osteoporosis may arise from an intrinsic genetic bone abnormality (primary osteoporosis) or an underlying medical condition and/or its treatment (secondary osteoporosis). The primary forms are relatively rare, whereas secondary forms of osteoporosis are increasingly observed in many chronic conditions [3]. Etiological factors responsible for osteoporosis secondary to chronic illness include immobility, pubertal delay and other hormonal disturbances, undernutrition and low body weight, inflammatory cytokines and glucocorticoid use [2,4].

Leukemia is the most common form of childhood malignancy, with acute lymphoblastic leukemia (ALL) accounting for approximately 75% of cases. With an overall survival rate approaching 80%, children with ALL have an excellent prognosis [3]. The two major skeletal complications of leukemia are osteoporosis and avascular necrosis [5].

Kaste *et al*. [6] revealed a bone mineral accumulation process during the period corresponding to onset of most childhood ALLs. Haddy *et al*. [7] noted that osteopenia/osteoporosis was observed in all phases of the disease: at diagnosis, during treatment, and throughout the post-treatment period for as long as 20 years. Among the findings that have been described are musculoskeletal pain, disturbed gait, fractures, kyphosis, lordosis, and growth failure. Pathologic fractures and vertebral collapses secondary to severe osteopenia (leukemic osteopathy) may occur [8].

Strauss *et al*. [5] reported a 28% 5-year cumulative fracture incidence in children with ALL. Rogalsky *et al*. [9] reported fractures in 25% of children with acute leukemia, 12% pathological, and 13% following trauma, during the course of their disease. Halton and Atkinson [10] reported that 39% of children with ALL had fractures by completion of their therapy.

An increased fracture frequency was also reported by van der Sluis *et al*. [11], who found the fracture rate in children with ALL to be six times that of healthy controls, up to 12 months following chemotherapy. Bone mass is often reduced at diagnosis in ALL and falls significantly during the first 6 months of chemotherapy [11,12]. Risk factors for the development of skeletal complications in ALL include poor nutrition, reduced mobility, impaired bone mineralization, older age at diagnosis and being of male gender [5,10].

About two-thirds of children with ALL will have had signs and symptoms of disease for less than 4 weeks at the time of their diagnosis. However, a history of some months is also compatible with the diagnosis of ALL. The first symptoms are usually nonspecific and include lethargy, unrelenting fatigue, bone pain or loss of appetite. More specific symptoms such as anemia, hemorrhage and infections are a consequence of lymphoblasts occupying the bone marrow and disturbing the residual normal hematopoiesis [8].

Bone pain is one of the initial symptoms in childhood ALLs that may result from direct leukemic infiltration of the periosteum, bone infarction, or expansion of the marrow cavity by leukemic cells. In normal hematopoiesis, hematopoietic stem cells (HSCs) are in balance with components of the hematopoietic microenvironment including osteoblastic cells, osteoclasts, mesenchymal cells, and vascular structures. In leukemia, invasion of leukemia cells results in osteopenia mediated by an expansion of osteoclasts causing increased bone reabsorption and a concomitant reduction of osteoblastic activity [13].

## Case presentation

A 7-year-old Asian Balinese boy was referred to our hospital because of persistent back pain over a 2-month period. The pain increased when he walked, but disappeared when he rested.

His respiratory rate was 26 breaths per minute, no cyanotic was noted, his heart rate was 96 beats per minute, and no grunting was noted. His axillaries temperature was 37°C. He had pale conjunctiva. He also had multiple lymphadenopathies to the right and left of both the abdominal and inguinal regions. The lymphadenopathies were mobile, with no sign of inflammation. There was no retraction on thorax region. We noticed an innocent murmur on auscultation, grade II/6. No enlargement of the liver or spleen was found. He had no rash, petechia nor edema on his extremities.

His weight was 18.5kg (<3rd percentile for his age, Centers for Disease Control and Prevention, CDC, 2000). His height was 118cm (his stature for his age puts him in the 10th to 25th percentile, CDC 2000), and head circumference was 50cm. His nutritional status was 84% according to Waterlow. According to his parents’ height (his father’s height was 173cm, his mother’s height was 165cm), his potential adult height would be around 167 to 184cm. His upper segment was 66cm and lower segment was 52cm. The upper–lower segment ratio was thus 1.27.

**Figure 1:**
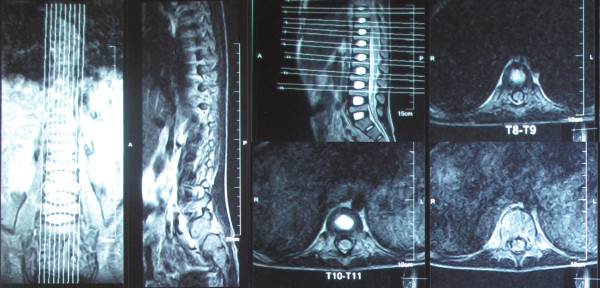
Magnetic resonance-imaging of the spine, 1 week after admission, revealed multiple wedge and biconcave compressions.

His initial complete blood count revealed a normal white blood cell (WBC) count (11.59×10^3^/uL), with low hemoglobin level (8.2g/dL). His mean corpuscular volume was 80.2fL. His platelet count was 41×10^3^/uL. A review of a peripheral blood smear showed a normochromic anemia with thrombocytopenia. His free thyroxine and thyroid-stimulating hormone levels were 1.56ng/dL and 3.24uIU/mL, respectively. His parathyroid hormone level was 14.09pg/mL. His calcium=9.10mg/dL, sodium=136.10mmol/L, potassium=4.33mmol/L, uric acid =4.1mg/dL, total bilirubin=0.22mg/dL, direct bilirubin=0.1mg/dL, alanine aminotransferase=21.99 U/L, aspartate aminotransferase=10.7 U/L, albumin=3.65g/dL, urea=13.2mg/dL and creatinine serum=0.41mg/dL. His serum iron was 133.9ug/dL, total iron binding capacity was 214ug/dL and ferritin was 282.7ng/mL.At first, the differential diagnosis was either osteoporosis with chronic infection or aplastic anemia. A tuberculin test was performed, and the result was negative. A posteroanterior and a lateral thorax radiograph showed multiple compressions of thoracic vertebrae. An anteroposterior pelvic radiograph showed osteoporosis. The thoracic vertebrae magnetic resonance imaging (MRI) showed multiple wedge and biconcave compressions of his left thoracal corpus vertebra and fatty marrow replacement in the osteoporotic bone marrow (Figure 1). He was treated daily with a 200mg oral dose of calcium for the osteoporosis.

His bone age, according to the S. Idell Pyle atlas, showed growth failure of his metacarpals, phalanges and sesamoid. His spine (0.482g/cm^2^) and total body BMD (0.805g/cm^2^) were normal, with a 97% age-match and a Z score of –0.5 SD.

**Figure 2:**
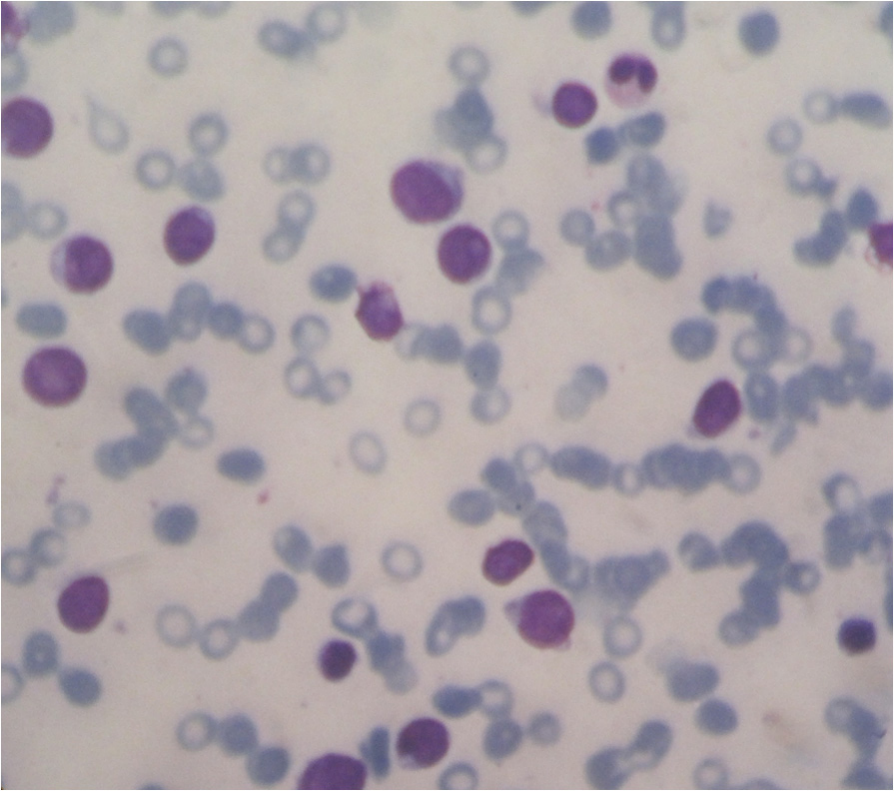
**Histological examination of a bone marrow aspiration, taken 1 month after admission, revealed acute lymphoblastic leukemia (L2).** The cytological feature revealed large-heterogeneous cells, with homogeneous nuclear chromatin, one or more nucleoli, and low activity in the erythroid, myeloid and megakaryocytic system.

One month later, his complete blood count showed pancytopenia (white blood count=3.54×10^3^/uL, hemoglobin=9.9g/dL and thrombocyte=37.2×10^3^/uL). His mean corpuscular volume was 86.4fL. He had a bone marrow aspiration and the results revealed ALL (L2). The sample was hypercellular, with round and oval nucleus shape, smooth homogeneous chromatin, and high nuclear–cytoplasmic ratio. There were low activities in his erythroid, myeloid and megakaryocytic system (Figure 2). We noticed a 50% lymphoblast cell infiltration with size variations. The immunophenotyping of peripheral blood revealed no dominant markers.

Based on the bone marrow aspiration, a diagnosis of ALL (L2) was made, excluding aplastic anemia and chronic infection anemia. He had serial chemotherapy for 109 weeks, with calcium and vitamin D supplements for the leukemia and secondary osteoporosis.

## Conclusions

In this case, the whole body BMD was decreased by 0.5 SD. Prospective studies have shown that a decrease in BMD by 1 SD is associated with a 1.5- to 3-fold increase in the relative risk of fracture [6,7]. The bone age assessment revealed a growth failure of the metacarpals, phalanges and sesamoid. Multiple compressions were seen in the posteroanterior and lateral thorax radiograph, the anteroposterior pelvic radiograph, and the thoracic vertebrae MRI, which signified osteoporosis.

For childhood ALLs, the WBC count may be low, normal, or increased. Our case had an initially normal WBC count, but it then dropped to 3.54×10^3^/uL. The hemoglobin level usually shows a moderate to marked reduction, with a normocytic normochromic red cell morphology. Blood smears for ALL with leucopenia usually show very few to no blasts, but when the WBC is greater than 10^4^/mm^3^, blasts are usually abundant. In our case the first blood smear showed normochromic anemia with thrombocytopenia. Both the blood smear and the immunophenotyping in this case revealed no blasts.

Leukemia is diagnosed when the bone marrow contains more than 25% blasts. Megakaryocytes are usually absent. The hallmark of the diagnosis of acute leukemia is blast cells, a relatively undifferentiated cell with diffusely distributed nuclear chromatin, one or more nucleoli, and basophilic cytoplasm. In this case, a bone marrow aspiration revealed hypercellularity and low activity in the erythroid, myeloid and megakaryocytic systems, with 50% lymphoblast cell infiltration.

There is a difference between normal hematopoiesis and leukemia in bone homeostasis. In normal hematopoiesis, HSCs are in balance with components of the hematopoietic microenvironment including osteoblastic cells, osteoclasts, mesenchymal cells, and vascular structures. In leukemia, invasion of leukemia cells results in osteopenia mediated by an expansion of osteoclasts causing increased bone reabsorption and a concomitant reduction of osteoblastic activity. The effect, if any, on other components of the HSC niche has yet to be determined [13].

Specialist studies such as immunophenotyping usually help in detailed leukemic subtype classification. Unfortunately, because of the leucopenia, the immunophenotype failed to reveal a dominant marker in this case, thus we were unable to classify the ALL.

## Consent

Written informed consent was obtained from the patient’s legal guardian(s) for publication of this case report and any accompanying images. A copy of the written consent is available for review by the Editor-in-Chief of this journal.

## Abbreviations

ALL: Acute lymphoblastic leukemia; BMD: Bone mass density; CDC: Centers for Disease Control and Prevention; HSC: Hematopoietic stem cell; MRI: Magnetic resonance imaging; SD: Standard deviation; WBC: White blood cell.

## Competing interests

The authors declare that they have no competing interests.

## Authors’ contributions

KA analyzed the patient data regarding the hematological disease. WBS interpreted the physical and laboratory examinations of the patient. MA evaluated the physical and radiological examinations regarding the osteoporosis disease. HS performed the histological examination of the bone marrow, and was a major contributor in writing the manuscript. All authors read and approved the final manuscript.
